# Dynamics of Gastrointestinal Activity and Ruminal Absorption of the Methane-Inhibitor, Nitroethane, in Cattle

**DOI:** 10.3389/fvets.2022.817270

**Published:** 2022-02-03

**Authors:** Aleksandar K. Božic, Hector Gutiérrez-Bañuelos, Agustin Corral-Luna, Gordon Carstens, Martha María Arévalos-Sánchez, Monserrath Félix-Portillo, Alberto Muro-Reyes, Claudio Arzola-Álvarez, Robin C. Anderson, Roger B. Harvey

**Affiliations:** ^1^Faculty of Agriculture, Department of Animal Science, University of Novi Sad, Novi Sad, Serbia; ^2^Unidad Académica de Medicina Veterinaria y Zootecnia, Universidad Autónoma de Zacatecas, Zacatecas, Mexico; ^3^Facultad de Zootecnia y Ecología, Universidad Autónoma de Chihuahua, Chihuahua, Mexico; ^4^Department of Animal Science, Texas A&M University, College Station, TX, United States; ^5^United States Department of Agriculture/Agricultural Research Service, Food and Feed Safety Research Unit, Southern Plains Agricultural Research Center, College Station, TX, United States

**Keywords:** methane emissions, fecal methane-producing activity, nitroethane, plasma, rumen methane-producing activity

## Abstract

Nitroethane is a potent methane-inhibitor for ruminants but little is known regarding simultaneous effects of repeated administration on pre- and post-gastric methane-producing activity and potential absorption and systemic accumulation of nitroethane in ruminants. Intraruminal administration of 120 mg nitroethane/kg body weight per day to Holstein cows (*n* = 2) over a 4-day period transiently reduced (*P* < 0.05) methane-producing activity of rumen fluid as much as 3.6-fold while concomitantly increasing (*P* < 0.05) methane-producing activity of feces by as much as 8.8-fold when compared to pre-treatment measurements. These observations suggest a bacteriostatic effect of nitroethane on ruminal methanogen populations resulting in increased passage of viable methanogens to the lower bovine gut. Ruminal VFA concentrations were also transiently affected by nitroethane administration (*P* < 0.05) reflecting adaptive changes in the rumen microbial populations. Mean (± SD) nitroethane concentrations in plasma of feedlot steers (*n* = 6/treatment) administered 80 or 160 mg nitroethane/kg body weight per day over a 7-day period were 0.12 ± 0.1 and 0.41 ± 0.1 μmol/mL 8 h after the initial administration indicating rapid absorption of nitroethane, with concentrations peaking 1 day after initiation of the 80 or 160 mg nitroethane/kg body weight per day treatments (0.38 ± 0.1 and 1.14 ± 0.1 μmol/mL, respectively). Plasma nitroethane concentrations declined thereafter to 0.25 ± 0.1 and 0.78 ± 0.3 and to 0.18 ± 0.1 and 0.44 ± 0.3 μmol/mL on days 2 and 7 for the 80 or 160 mg nitroethane/kg body weight per day treatment groups, respectively, indicating decreased absorption due to increased ruminal nitroethane degradation or to more rapid excretion of the compound.

## Introduction

The production of methane by methanogens within the rumen represents a digestive inefficiency for the host that results in the loss of up to 12% of the gross energy consumed by the animal and contributes nearly 27% of the total United States' emissions of this potent greenhouse gas (1, 2). Despite its contribution to the loss of assimilable carbon and energy to the host, the production of methane within the rumen performs a valuable ecological function for the microbes inhabiting the rumen by maintaining hydrogen concentrations below 1 kPa (3). Hydrogen concentrations higher than 1 kPa inhibit microbial hydrogen gas-evolving hydrogenase activity thereby precluding an important method for re-oxidization of reduced nucleotides produced during fermentation and preventing re-entry of the re-oxidized electron carriers into fermentative pathways (3). Consequently, in the search for interventions to inhibit rumen methane emissions, microbiologists have often sought strategies that may promote flow of reducing substrates produced during fermentation away from methanogenesis and into alternative energetically favorable electron sinks (4). The administration of supplemental nitrate (${\text{NO}}_{3}^{-}$) to the diets of ruminants is one of few alternative electron strategies currently being investigated (5–7). The advantages of nitrate as an alternative electron acceptor are that its reduction to nitrogen gas or ammonia within the rumen microbial community is energetically favorable and stoichiometry comparable to methanogenesis in terms of electron consumption, consuming 6 to 8 electrons for complete reduction of nitrate to nitrogen or ammonia, respectively (8, 9). This technology is not yet commercially available, however, and awaits the development of practical, producer-friendly technologies to manage risks of animal toxicity associated with excessive accumulations of nitrite, a toxic metabolic intermediate of nitrate metabolism within the rumen (8, 9). Feeding nitrate requires diets to have adequate amounts of readily fermentable substrate to provide sufficient reducing power to sustain dissimilatory nitrate reduction (8, 9). It is known also that ruminants can be gradually adapted to high nitrate-containing diets to enrich *in situ* populations of nitrate- and nitrite-metabolizing bacteria (8, 9). Livestock producers, however, may be reluctant to implement an adaption strategy because it adds extra work and time during to their production cycles. Recently, another strategy being investigated seeks to reduce risks of nitrite toxicity during high nitrate supplementation *via* co-administration of a hyper-active nitrite-metabolizing ruminal bacterium as a direct fed microbial to ensure rapid metabolism and detoxification of nitrite produced in the rumen during metabolism of fed nitrate (10, 11).

Earlier studies showed that the naturally occurring phytotoxin, 3-nitro-1-propionate, was a potent inhibitor of ruminal methane production (12, 13). In this case, 3-nitro-1-propionate and another related naturally occurring phytotoxin, 3-nitro-1-propanol were found to exert direct inhibitory activity against methanogens in rumen microbial populations having no prior exposure to these nitrocompounds. However, the microbial populations exhibited the ability to adapt to the nitrocompounds by using them as alternative electron acceptors (12). This adaptation within the rumen microbial community was hypothesized to occur due to a 1,000–1,000,000-fold enrichment in numbers of an obligate anaerobic-respiring nitro-utilizing bacterium reported to normally be present at <10^3^ organisms/mL within the rumen (14). *Denitrobacterium detoxificans*, appears at present to be the only known ruminal bacteria to exhibit appreciable nitroalkane-metabolizing activity (15, 16). Other xenobiotic short chain nitrocompounds such as nitroethane, 2-nitroethanol, 2-nitro-1-propanol, dimethyl-2-nitroglutarate, 2-nitro-methyl-propionate, and ethyl-2-nitroacetate have also been shown to inhibit ruminal methane production *in vitro* (17–22). In the case of 3-nitro-1-propionate, 3-nitro-1-propanol and nitroethane, their reduction to their respective amines β-alanine, 3-amino-1-propanol and ethylamine has been demonstrated (23, 24). The fate of the other tested nitrocompounds has yet to be conclusively elucidated.

*In vivo* studies examining the methane-reducing potential of the naturally occurring 3-nitro-1-propionate and 3-nitro-1-propanol have not yet been undertaken; however, *in vivo* studies have examined the effects of nitroethane, 2-nitro-1-propanol or 2-nitroethanol administration to cattle or sheep (25–29) with all indicating efficacious decreases in ruminal methane-producing activity or rumen methane emission. While published reports exist for absorption of nitroethane as well as other short chain nitroalkanes in non-ruminants, mainly for investigations relating to occupational exposures to nitroalkanes as reviewed by Smith and Anderson (30), much less is known regarding nitroethane absorption in cattle or sheep. The objectives of the two separate trials of the work presented here were to fill in knowledge gaps pertaining to oral nitroalkane administration by assessing the potential impact of repeated nitroethane administration on pre- and post-gastric methane-producing activity as well as on potential absorption or systemic accumulation of nitroethane in ruminants.

## Materials and Methods

Rearing, care, and use of cattle in the following studies was approved by the USDA/ARS Southern Plains Agricultural Research Center's Animal Care and Use Committee. In the first study, two non-lactating rumen-cannulated Holstein–Friesian cows (each ~500 kg live body weight) maintained on a predominantly rye grass pasture were intra-ruminally administered nitroethane (CH_3_CH_2_NO_2_) at 120 mg/kg body weight per day in two equal sized portions (0800 and 1600) over a 4-day administration period which was followed by a subsequent 2-day post-administration period. The nitroethane treatments were administered as a sodium salt solution prepared as described by Majak et al. (31). Briefly, 131 mL nitroethane (density = 1.05 g/mL) was mixed with 345 mL 5 N sodium hydroxide, stirred vigorously for 15 min and then combined with 665 mL 0.4 M sodium phosphate buffer (pH 6.5) to achieve a 125 g nitroethane/liter solution. Rumen fluid was collected from contents obtained through the rumen cannula and strained through a nylon paint strainer (32) and fecal contents were collected *via* rectal palpation. Collection times were 2 h before the first nitroethane administration and at 2, 8, and 16 h and 1, 2, 3, and 4 days after the initial pre-administration sampling and again at 5 and 6 days after the initial sampling. The latter two samplings were 24 and 48 h after the last nitroethane administration. For determination of methane-producing activity in collected rumen fluid and feces, 5 mL portions of the freshly collected rumen fluid or 2 g portions of freshly collected feces were distributed, in triplicate for each cow and each sampling period, to 18 mm × 150 mm crimp top glass tubes preloaded with 0.2 g ground alfalfa. The tube contents were then mixed with 5 or 8 mL anaerobic buffer, respectively, added while under a continuous flow of hydrogen:carbon dioxide gas (50:50) (26). The anaerobic buffer was that of Bryant and Burkey (33) modified to contain 16 mM sodium formate. Tubes were capped immediately after addition of buffer and then incubated at 39°C for 3 h. Amounts of methane produced in the tubes at the end of incubation were measured by gas chromatography as described by Allison et al. (34) and methane-producing activity was calculated as the net amount of methane produced divided by 3 h and is expressed as μmol methane produced/g rumen fluid or feces per h with rumen fluid and feces assigned a density of 1 g/mL. Portions of rumen contents were also used for determination of ruminal volatile fatty acid (VFA) concentrations which were measured *via* gas chromatography (35). Concentrations of VFA were not measured in fecal collections.

In a second study, conducted to investigate the metabolism and absorption of nitroethane, blood samples were collected *via* jugular venipuncture from 18 steers (403 ± 26 kg BW; mean ± SD) fed a concentrate diet. The steers were offered a diet consisting of 50% dry rolled corn, 25% chopped alfalfa, 13% cotton seed hulls, 7% molasses, 3% soybean meal (49% crude protein), and 2% premix, containing vitamins, urea, limestone, and salt (26). Treatments (0, 80, or 160 mg nitroethane/kg BW) were administered individually to steers (6 steers/treatment) each reared in separate pens by oral gavage twice daily (0800 and 1630) for 14 days using an oral drench gun. Results pertaining to methane-producing and nitroethane-degrading activity in ruminal and fecal contents collected from these were reported earlier (26). Blood samples were collected into heparinized tubes prior to each mornings' treatment administration on days 0, 1, 2, and 7 of the study. Blood samples were also collected 2 h after the initial treatment administration. All blood samples were frozen (−20°C) until analysis for nitroethane concentrations which were determined colorimetrically (36) as well as by high performance liquid chromatography (37). Samples were clarified prior to analysis using zinc sulfate-sodium hydroxide precipitation method (38).

Associations between rumen and fecal methane-producing activity were assessed by Pearson correlation. Associations between plasma nitroethane measurements determined colorimetrically or by high performance liquid chromatography were similarly compared using Pearson correlation. To test for differences in ruminal and fecal methane-producing activity before, during, and after cessation of oral nitroethane treatment, each animal served as its own control and rates of methane-producing activity as well as ruminal concentrations of VFA were compared across each sampling day using a repeated measures analysis of variance, with a fixed effect of day, and an LSD multiple comparison of means. In the second study, tests for effect of treatment on plasma concentrations of nitroethane were conducted using a repeated measures analysis of variance, with fixed effects of day and dose of nitroethane, and an LSD multiple comparison of means. All analyses were conducted using Statistix9 Analytical Software (Tallahassee, FL). Significance was declared at *P* ≤ 0.05.

## Results and Discussion

**Figure 1:**
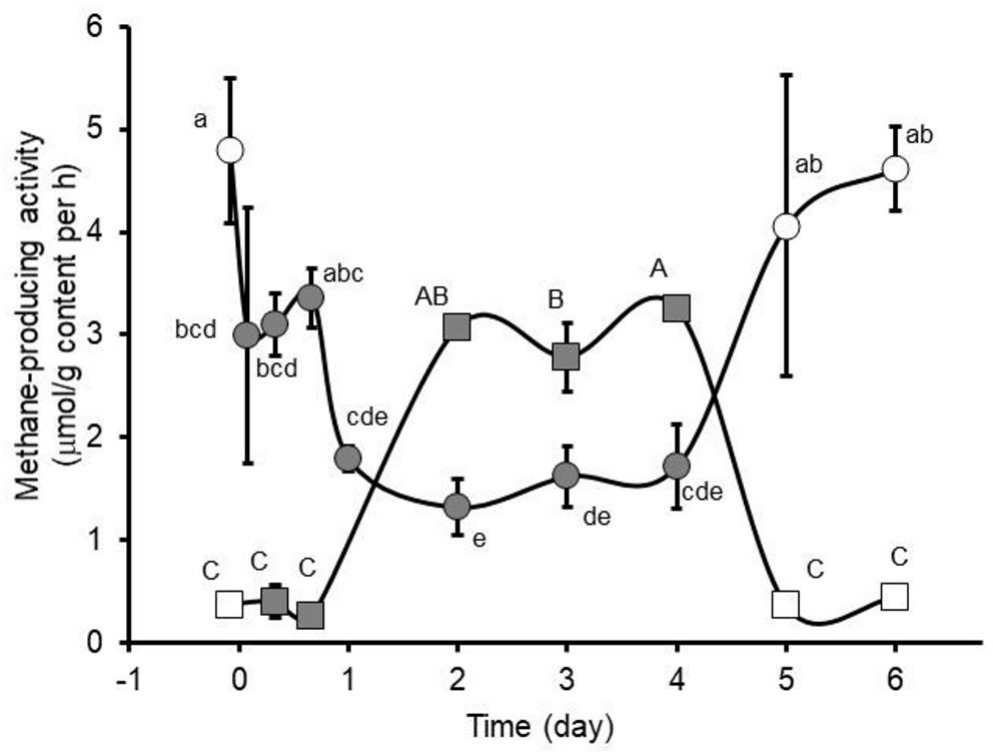
Ruminal (circles) and fecal (squares) methane-producing activity measured in pastured cows during pre-treatment, twice daily intraruminal administration (closed symbols), and post-treatment with 160 mg nitroethane/kg body weight (BW). Pre- and post-treatment values are presented with open symbols. Main effects of day were observed (*P* = 0.0057 and <0.0001, respectively) on ruminal and fecal methane-producing activity. Means (± SD from *n* = 2 cows) with unlike lower-case (ruminal) and upper-case (fecal) letters differ at *P* < 0.05.

Consistent with earlier reports (25–27), intraruminal administration of 120 mg nitroethane/kg body weight decreased (*P* < 0.05) methane-producing activity of the rumen fluid by as much as 72% compared to pre-treatment activity. Unlike the earlier studies, the present study examined effects of intraruminal nitroethane administration on methane-producing activity of collected feces and found up to a 9-fold increase (*P* < 0.05) in methane-producing activity of the feces that was inversely correlated (Pearson's correlation coefficient = −0.84, *P* < 0.0001) with the observed decrease in methane-producing activity of the rumen fluid. Moreover, results revealed that methane-producing activities of the rumen fluid and feces returned to pre-treatment levels upon cessation of nitroethane administration indicating that nitroethane needed to be present to sustain inhibition of methanogenesis (Figure 1). These assays, which were conducted with non-limiting amount of substrate, indirectly reflect numbers of methanogens and thus it seems reasonable to conclude that intraruminal nitroethane administration bacteriostatically caused a decrease in numbers of methanogens within the rumen. The concomitant increase in methane-producing activity of the feces suggests the passage of surviving methanogens through the abomasum to the lower gut. Whether or not the viable methanogens arriving to the lower gut of these cattle were able to produce appreciable amounts of methane in the large intestine is not discernable from the data available here but further research may be warranted to investigate the impact of such a possibility on overall efficacy of this or similar bacteriostatic methane-reduction technologies. More recently, Zhang et al. (39) reported from *in vitro* studies that nitroethane, 2-nitroethanol and 2-nitro-1-propanol significantly decreased numbers of methanogens during *in vitro* fermentation of freshly collected rumen fluid *via* inhibition of expression of coenzymes contributing to methanogenesis. In their study, however, they reported that while populations of *Methanobacteriales* were decreased they were not eliminated even after 72 h incubation (39). Populations of *Methanomicrobiales* and *Methanococcales* were also decreased by nitrocompound treatment during the first 12 h of incubation but by 24 h of incubation the abundance of these methanogens had decreased to very low numbers or undetectable levels in the treated as well as untreated incubations (39). Anderson et al. (18) had suggested that nitroethane and other tested nitroalkanes inhibited methanogenesis by inhibiting the oxidation of hydrogen and formate, the major reducing substrates for methane production. Zhang et al. (39) reported that the decrease in methanogen numbers observed in their *in vitro* study was associated with decreased expression of *mcr*A encoding methyl-coenzyme M reductase as well as amounts of coenzymes *F*_420_ and *F*_430_, all important contributors to methanogenesis. It is not clear from the results reported by Zhang et al. (39) if the nitroalkanes actually inhibited the activity of the co-enzymes, but the methane-inhibiting activity of 3-nitrooxypropanol investigated by others was reported to be due to targeted inhibition of methyl-coenzyme M reductase activity *via* binding to the coenzyme active site (40).

**Figure 2:**
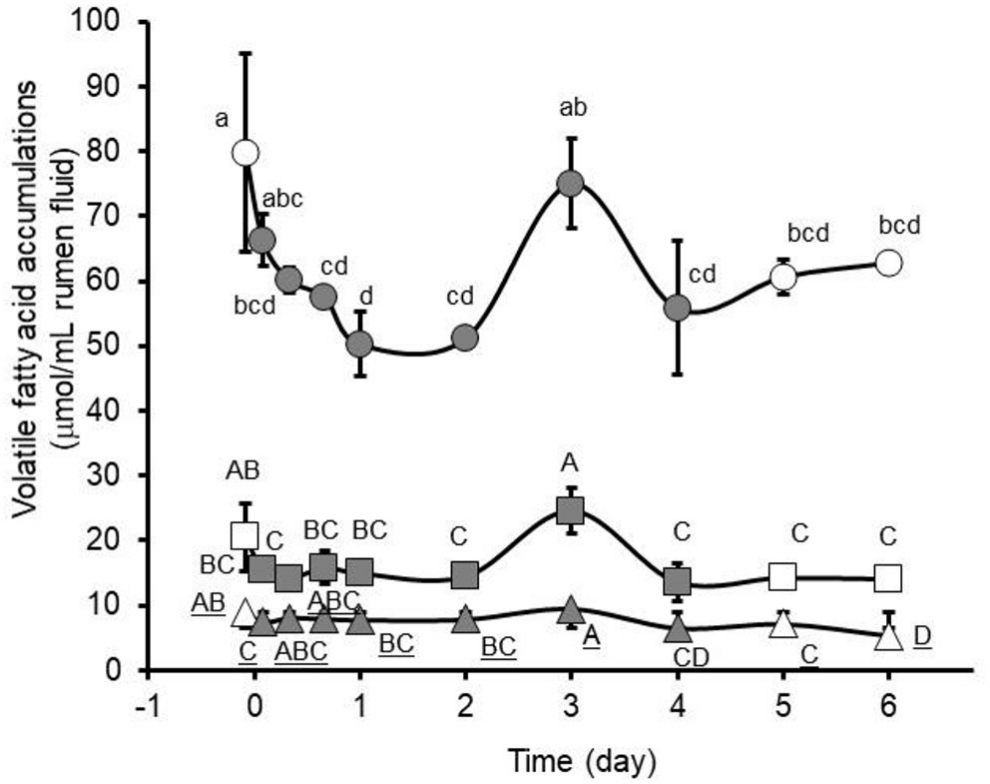
Ruminal accumulations of acetate (circles), propionate (squares), or butyrate (triangles) observed in pastured cows during pre-treatment, twice daily intraruminal administration (closed symbols), and post-treatment with 160 mg nitroethane/kg body weight (BW). Pre- and post-treatment values are presented with open symbols. Main effects of day were observed (*P* = 0.0324, 0.0243, and <0.0089, respectively) on ruminal acetate, propionate and butyrate concentrations. Means (± SD from *n* = 2 cows) with unlike lower-case (acetate), upper-case (propionate), and underlined upper-case letters (butyrate) differ at *P* < 0.05.

Ruminal accumulations of VFA in this study were affected (*P* < 0.05) by nitroethane administration (Figure 2). In this case, concentrations of the acetate, propionate and butyrate gradually decreased from pre-treatment concentrations, although not necessarily significantly, during the first 2 days of nitroethane administration (Figure 2). Concentrations of acetate, propionate and butyrate then abruptly increased (*P* < 0.05) in the rumen to near pre-treatment concentrations on the 3rd day of nitroethane administration which was then followed again by a decrease (*P* < 0.05) in concentrations on the fourth (last) day of nitroethane administration. This pattern of VFA concentration potentially reflects an unstable change within the rumen microbial population due to nitroethane treatment. For instance, it is possible that the microbial population may not have had sufficient time to adapt to the twice daily intraruminal nitroethane administration during the first 2 days of the study thereby allowing nitroethane concentrations to accumulate to levels inhibitory to fermentative bacteria. It is known that *Denitrobacterium detoxificans*, at present the only ruminal bacteria known to express appreciable nitroalkane-metabolizing activity, can be enriched in numbers in the presence of nitroethane or other suitable acceptors yet the sustainability of this bacterium at high population levels is not yet certain. For instance, the abrupt increase in VFA concentrations within the rumen fluid collected on the 3rd day of nitroethane administration may reflect an *in situ* enrichment in numbers of *D. detoxificans*, but maintenance of this bacteria at numbers higher than normally present in the rumen may have been limited in this case due to exhaustive consumption of available substrates needed for growth.

**Figure 3:**
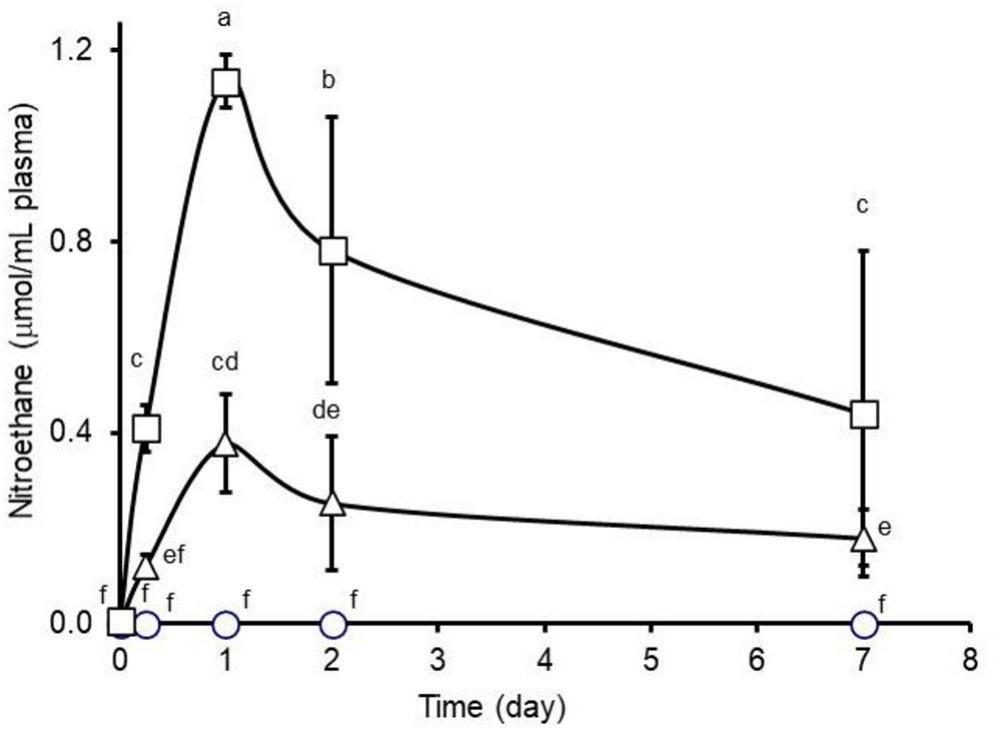
Plasma nitroethane concentrations observed during 7-day oral administration (twice daily, 0800 and 1600) of 0 (circles), 1.1 (triangles), or 2.2 (squares) μmol nitroethane/kg body weight (BW) per day to feedlot steers. An interaction between treatment and day was observed (*P* = 0.0001). Means (± SD from *n* = 6 steers per treatment) with unlike lower-case letters differ at *P* < 0.05.

In support of increased ruminal metabolism, rates of ruminal nitroethane-degrading activity in contents collected from steers orally administered nitroethane over a 2-week period were reported to be >2.5-fold higher than rates observed pre-treatment or in steers administered no nitroethane (26). This observation suggests, as discussed above, the enrichment of nitroethane-degrading bacteria in the rumen of steers administered nitroethane and is consistent with earlier reports of rumen enrichment of the nitroalkane-metabolizing activity (14, 31, 41). Conversely, rates of fecal nitroethane-degrading activity were reported to be unaffected by nitroethane treatment (26) indicating that nitroethane was not present at high enough concentrations in the lower gut to affect a similar enrichment of nitroethane-degrading bacteria in these steers. Additionally, mean (± SD) nitroethane concentrations in plasma collected from these steers and analyzed in the present study were 0.12 ± 0.1 and 0.41 ± 0.1 μmol/mL for steers administered 80 or 160 mg nitroethane/kg BW per day, respectively, when measured 6 h after the start of nitroethane treatment indicating rapid absorption of nitroethane. Plasma nitroethane concentrations peaked 1 day after initiation of the 80 or 160 mg nitroethane/kg BW per day treatments (0.38 ± 0.1 and 1.14 ± 0.1 μmol/mL, respectively) (Figure 3). Assuming a 60 mL blood volume/kg live body weight (42), total daily administration of nitroethane would be equivalent 2.1 and 4.3% the total daily administration of nitroethane (32,240 and 64,480 mg/steer, respectively). These estimates compare favorably to peak absorption of 1.8% 3-nitro-1-propanol administered as a single dose of 20 mg/kg body weight to cattle (43). Plasma nitroethane concentrations declined thereafter to 0.25 ± 0.1 and 0.78 ± 0.3 and to 0.18 ± 0.1 and 0.44 ± 0.3 μmol/mL on days 2 and 7 for the 80 or 160 mg nitroethane/kg body weight per day treatment groups, respectively. Plasma nitroethane concentrations presented above were determined colorimetrically but these values agreed well with those determined by high performance liquid chromatography (not shown) with Pearson's correlation coefficient being 0.96 thus supporting the findings of the colorimetric analysis. The gradual decrease in plasma nitroethane concentrations during successive sampling days supports the suggestion that the reported increase in rates of ruminal nitroethane metabolism may have contributed to lesser amounts of nitroethane being available for absorption. As expected, nitroethane was not detected in plasma samples collected from untreated steers. Absorption of the naturally occurring 3-nitro-1-propanol and 3-nitro-1-propionic acid from the bovine and ovine rumen occurs rapidly, accumulating in plasma mainly as 3-nitro-1-propionic acid as the nitroalcohol is quickly converted to the nitroacid by hepatic alcohol dehydrogenase (43, 44). As alluded to earlier, it is generally thought that strategies that enhance rates of ruminal metabolism of the natural-occurring nitrocompounds can confer protection against poisoning by decreasing amounts of toxin available for absorption (31, 41).

Results from the present study provide new information pertaining to the pre- and post-gastric effects of ruminal nitroethane administration on methane-producing microbial populations in cattle as well as new information regarding absorption of nitroethane during 7-day nitroethane treatment. From a practical perspective, results from a limited number of *in vivo* studies indicates that application of nitroethane as well as 2-nitroethanol and 2-nitro-1-propanol (25–27, 29) as ruminal methane inhibitors may achieve near equivalent efficacy in methane abatement as the 3-nitroxycompound current commercially available in certain markets (45, 46), albeit the latter at considerable lower doses. An additional advantage of the nitroalkanes is that the consumption of electrons during their reduction may help preserve energetic efficiencies associated with inter-species hydrogen transfer reactions similar to that achieved with supplemental nitrate feeding (8, 9). Moreover, the biodegradability of the methane inhibitors may be advantageous as the absence of methane-inhibiting activity along with the presence of viable methanogen populations in animal manure would be compatible with biotechnological waste treatment technologies intending to produce methane as an economically valuable renewable energy source. For instance, contrary to methane production in the rumen, which contributes a source of carbon to the atmosphere with a global warming potential 28-times greater than that of carbon dioxide (47), the production of methane as a renewable energy source provides an opportunity to recycle carbon already present within the contemporary carbon cycle. Conceptually, the recycling of carbon already present in the earth's atmosphere can decrease the need to extract an equivalent amount of methane from fossil fuel reserves sequestered deep within the earth thereby yielding a renewable energy source of potentially equal or greater economic and societal value.

A disadvantage, however, of xenobiotic nitrocompounds is their need to undergo extensive testing to address toxicity and safety concerns and obtain regulatory approval. In the case of these primary nitroalkanes and substituted nitroalkanes, toxicity concerns dealing with respiratory irritation will need to be addressed (30). However, chronic exposure of rats to 100 or 200 ppm nitroethane for 7 h per day, 5 days per week for 2 years did not result in any measurable adverse hematological or biological effects as reflected by organ weights and clinical chemistry (48). Additionally, reports from *in vivo* animal studies investigating the methane inhibiting activity of nitroethane observed no apparent adverse effects on animal well-being (25–27, 29) which suggests concerns regarding toxicity may not necessarily be exclusionary. Moreover, at least in the case of nitroethane and 2-nitropropanol, it is unclear if their presumed reduction products, ethylamine and aminopropanol, may be of nutritional value to the ruminant host. It has been reported that ethylamine can serve as a precursor for microbial l-theanine synthesis (49, 50) but whether this may occur within the rumen is not known. Another important disadvantage of the non-natural compounds is that they may not be compatible with organic production segments of the respective meat and dairy industries. In this regard, application of naturally occurring nitrocompounds may be attractive for organic segments of the industries, providing of course that toxicity issues concerning 3-nitro-1-propionic acid, and especially 3-nitro-1-propanol, can be satisfactorily controlled. Whereas, the ultimate fate of 3-amino-1-propanol in the rumen has yet to be determined, β-alanine, a non-essential amino acid, is known to be metabolized by rumen microbes to products that can be used by the host as sources of carbon, nitrogen and energy (23, 51). Additionally, it seems reasonable that combined administration of natural 3-nitro-1-propionate sources with a probiotic-preparation of the naturally occurring ruminal nitro-reducing microbe *D. detoxificans* to optimize electron consumption during metabolism of 3-nitro-1-propionate to β-alanine (six electron reduction) may effectively minimize toxicity issues. Results from an early study has indicated that inoculation of cattle grazing pasture containing 3-nitro-1-propionate-accumulating forage, another legume known to accumulate high levels of 3-nitro-1-propionate, with laboratory-grown *D. detoxificans* provided measurable protection to the animals (52) but clearly, further studies are warranted to further characterize and optimize the methane-abatement potential of these various nitrocompounds.

## Data Availability Statement

The raw data supporting the conclusions of this article will be made available by the authors, without undue reservation.

## Ethics Statement

The animal study was reviewed and approved by United States Department of Agriculture, Agricultural Research Service, Southern Plains Agricultural Research Center Animal Care and Use Committee, College Station, TX.

## Author Contributions

AB, HG-B, RCA, and RBH contributed equally to the design and planning of the studies. AB, HG-B, AC-L, GC, MA-S, MF-P, AM-R, CA-Á, and RCA contributed to the conduct of the study, data analysis, interpretation of results, and writing of the paper. All authors have read and approved the final version of the manuscript.

## Funding

This work was funded by the United States Department of Agriculture.

## Conflict of Interest

The authors declare that the research was conducted in the absence of any commercial or financial relationships that could be construed as a potential conflict of interest.

## Publisher's Note

All claims expressed in this article are solely those of the authors and do not necessarily represent those of their affiliated organizations, or those of the publisher, the editors and the reviewers. Any product that may be evaluated in this article, or claim that may be made by its manufacturer, is not guaranteed or endorsed by the publisher.

## References

[1] Johnson KA Johnson DE. Methane emissions from cattle. J Anim Sci. (1995) 73:2483–92. 10.2527/1995.7382483x 8567486

[2] EPA; Environmental Protection Agency. Inventory of U.S. Greenhouse Gas Emissions Sinks: 1990-2019. United States Environmental Protection Agency. (2021). Available online at: https://www.epa.gov/ghgemissions/inventory-us-greenhouse-gas-emissions-and-sinks-1990-2019 (accessed September 27, 2021).

[3] Miller TL. The ecology of methane production and hydrogen sinks in the rumen. In: Engelhardt WV, Leonhard-Marek S, Breves G, Giesecke D, editors. Ruminant Physiology: Digestion, Metabolism, Growth and Reproduction. Berlin: Ferdinand Enke Verlag (1995). p. 317–31.

[4] McAllister TA Newbold CJ. Redirecting rumen fermentation to reduce methanogenesis. Aust J Exp Agric. (2008) 48:7–13. 10.1071/EA0721828948418

[5] van Zijderveld SM Gerrits WJJJ Dijkstra J Newbold JR Hulshof RBA Perdok HB. Persistency of methane mitigation by dietary nitrate supplementation in dairy cows. J Dairy Sci. (2011) 94:4028–38. 10.3168/jds.2011-4236 21787938

[6] Hulshof RB Berndt AA Gerrits WJJ Dijkstra J Zijderveld V Newbold MJR. Dietary nitrate supplementation reduces methane emission in beef cattle fed sugarcane-based diets. J Anim Sci. (2012) 90:2317–23. 10.2527/jas.2011-4209 22287674

[7] Lin M Guo WS Meng QX Stevenson DM Weimer PJ Schaefer DM. Changes in rumen bacterial community composition in steers in response to dietary nitrate. Appl Microbiol Biotechnol. (2013) 97:8719–27. 10.1007/s00253-013-5143-z 23955503

[8] Latham EA Anderson RC Pinchak WE Nisbet DJ. Insights on alterations to the rumen ecosystem by nitrate and nitrocompounds. Front Microbiol. (2016) 7:228. 10.3389/fmicb.2016.00228 26973609 PMC4777734

[9] Yang C Rooke JA Cabeza I Wallace RJ. Nitrate and inhibition of ruminal methanogenesis: microbial ecology, obstacles, and opportunities for lowering methane emissions from ruminant livestock. Front Microbiol. (2016) 7:132. 10.3389/fmicb.2016.00132 26904008 PMC4751266

[10] Latham EA Pinchak WE Trachsel J Allen HK Callaway TR Nisbet DJ. Isolation, characterization and strain selection of a Paenibacillus species for use as a probiotic to aid in ruminal methane mitigation, nitrate/nitrite detoxification and food safety. Bioresour Technol. (2018) 263:358–64. 10.1016/j.biortech.2018.04.116 29758485

[11] Latham EA Pinchak WE Trachsel J Allen HK Callaway TR Nisbet DJ. Paenibacillus 79R4, a potential rumen probiotic to enhance nitrite detoxification and methane mitigation in nitrate-treated ruminants. Sci Total Environ. (2019) 671:324–8. 10.1016/j.scitotenv.2019.03.390 30933788

[12] Anderson RC Rasmussen MA. Use of a novel nitrotoxin-metabolizing bacterium to reduce ruminal methane production. Bioresour Technol. (1998) 64:89–95. 10.1016/S0960-8524(97)00184-3

[13] Anderson RC Ripley LH Bowman JGP Callaway TR Genovese KJ Beier RC. Ruminal fermentation of anti-methanogenic nitrate- and nitro-containing forages *in vitro*. Front Vet Sci. (2016) 3:62. 10.3389/fvets.2016.00062 27563646 PMC4980585

[14] Anderson RC Rasmussen MA Allison MJ. Enrichment and isolation of a nitropropanol metabolizing bacterium from the rumen. Appl Environ Microbiol. (1996) 62:3885–6. 10.1128/aem.62.10.3885-3886.1996 8837447 PMC168200

[15] Anderson RC Rasmussen MA DiSpirito AA Allison MJ. Characteristics of a nitropropanol-metabolizing bacterium from the rumen. Can J Microbiol. (1997) 43:617–24. 10.1139/m97-088 9246740

[16] Anderson RC Rasmussen MA Jensen NS Allison MJ. Denitrobacterium detoxificans gen. nov., sp. nov., a ruminal bacterium that respires on nitrocompounds. Int J Syst Evol Microbiol. (2000) 50:633–8. 10.1099/00207713-50-2-633 10758869

[17] Anderson RC Callaway TR Van Kessel JS Jung YS Edrington TS Nisbet DJ. Effect of select nitrocompounds on ruminal fermentation; an initial look at their potential to reduce economic and environmental costs associated with ruminal methanogenesis. Bioresour Technol. (2003) 90:59–63. 10.1016/S0960-8524(03)00086-5 12835058

[18] Anderson RC Krueger NA Stanton TB Callaway TR Edrington TS Harvey RB. Effects of select nitrocompounds on *in vitro* ruminal fermentation during conditions of limiting or excess added reductant. Bioresour Technol. (2008) 99:8655–61. 10.1016/j.biortech.2008.04.064 18538564

[19] Anderson RC Huwe JK Smith DJ Stanton TB Krueger NA Callaway TR. Effect of nitroethane, dimethyl-2-nitroglutarate and 2-nitro-methyl-propionate on ruminal methane production and hydrogen balance *in vitro*. Bioresour Technol. (2010) 101:5345–9. 10.1016/j.biortech.2009.11.108 20194018

[20] Gutierrez-Bañuelos H Anderson RC Carstens GE Tedeschi LO Pinchak WE Cabrera-Diaz E. Effects of nitroethane and monensin on ruminal fluid fermentation characteristics and nitrocompound-metabolizing bacterial populations. J Agric Food Chem. (2008) 56:4650–8. 10.1021/jf800756c 18491914

[21] Božic AK Anderson RC Carstens GE Ricke SC Callaway TR Yokoyama MT. Effects of the methane-inhibitors nitrate, nitroethane, lauric acid, Lauricidin^®^ and the Hawaiian marine algae, Chaetoceros, on ruminal fermentation *in vitro*. Bioresour Technol. (2009) 100:4017–25. 10.1016/j.biortech.2008.12.061 19362827

[22] Ochoa García P Anderson RC Arévalos Sánchez MM Rodríguez Almeida FA Félix Portillo M Muro Reyes A. *Astragallus mollissimus* plant extract: a strategy to reduce ruminal methanogenesis. Trop Anim Health Prod. (2021) 53:436. 10.1007/s11250-021-02882-1 34401959

[23] Anderson RC Rasmussen MA Allison MJ. Metabolism of the plant toxins nitropropionic acid and nitropropanol by ruminal microorganisms. Appl Environ Microbiol. (1993) 59:3056–61. 10.1128/aem.59.9.3056-3061.1993 8215375 PMC182406

[24] Ruiz-Barrera O Anderson RC Hume ME Corrales-Millan J Castillo-Castillo Y Corral-Luna A. Short chain nitrocompounds as a treatment of layer hen manure and litter; effects on *in vitro* survivability of Salmonella, generic coli E, nitrogen metabolism. J Environ Sci Health Part B. (2017) 52:23–9. 10.1080/03601234.2016.1224698 27628961

[25] Anderson RC Carstens GE Miller RK Callaway TR Schultz CL Edrington TS. Effect of oral nitroethane and 2-nitropropanol administration on methane-producing activity and volatile fatty acid production in the ovine rumen. Bioresour Technol. (2006) 97:2421–6. 10.1016/j.biortech.2005.10.013 16303299

[26] Gutierrez-Bañuelos H Anderson RC Carstens GE Slay LJ Ramlachan N Horrocks SM. Zoonotic bacterial populations, gut fermentation characteristics and methane production in feedlot steers during oral nitroethane treatment and after the feeding of an experimental chlorate product. Anaerobe. (2007) 13:21–31. 10.1016/j.anaerobe.2006.11.002 17208022

[27] Brown EG Anderson RC Carstens GE Gutierrez-Bañuelos H McReynolds JL Slay LJ. Effects of oral nitroethane on enteric methane emissions and ruminal fermentation in cattle. Anim Feed Sci Technol. (2011) 166–7:275–81. 10.1016/j.anifeedsci.2011.04.017

[28] Zhang ZW Wang YL Chen YY Wang WK Zhang LT Luo HL. Nitroethanol in comparison with monensin exhibits greater feed efficiency through inhibiting rumen methanogenesis more efficiently and persistently in feedlotting lambs. Animals (Basel). (2019) 9:784. 10.3390/ani9100784 31614547 PMC6826695

[29] Zhang ZW Wang YL Chen YY Zhang LT Zhang YJ Liu YQ. The dietary supplemental effect of nitroethanol in comparison with monensin on methane emission, growth performance and carcass characteristics in female lambs. Animals (Basel). (2021) 11:327. 10.3390/ani11020327 33525565 PMC7911303

[30] Smith DJ Anderson RC. Toxicity and metabolism of nitroalkanes and substituted nitroalkanes. J Agric Food Chem. (2013) 61:763–79. 10.1021/jf3039583 23294468

[31] Majak W Cheng K-J Hall JW. Enhanced degradation of 3-nitropropanol by ruminal microorganisms. J Animal Sci. (1986) 62:1072–80. 10.2527/jas1986.6241072x 3710925

[32] Leyendecker SA Callaway TR Anderson RC Nisbet DJ. Technical note on a much simplified method of collecting ruminal fluid using a nylon paint strainer. J Sci Food Agric. (2004) 84:387–9. 10.1002/jsfa.167325855820

[33] Bryant MP Burkey LA. Cultural methods and some characteristics of some of the more numerous groups of bacteria in the bovine rumen. J Dairy Sci. (1953) 36:205–17. 10.3168/jds.S0022-0302(53)91482-9

[34] Allison MJ Mayberry WR McSweeney CS Stahl DA. *Synergistes jonessi*, gen. nov., sp. nov.: a ruminal bacterium that degrades toxic pyridinediols. Syst Appl Microbiol. (1992) 15:522–9. 10.1016/S0723-2020(11)80111-6

[35] Hinton A Corrier DE Spates GE Norman JO Ziprin RL Beier RC. Biological control of *Salmonella typhimurium* in young chickens. Avian Dis. (1990) 34:626–33. 10.2307/1591255 2241691

[36] Majak W Clark LJ. Metabolism of aliphatic nitro compounds in bovine rumen fluid. Can J Anim Sci. (1980) 60:319–25. 10.4141/cjas80-041

[37] Muir AD Majak W. Quantitative determination of 3-nitropropionic acid and 3-nitropropanol in plasma by HPLC. Toxicol Lett. (1984) 20:133–6. 10.1016/0378-4274(84)90137-1 6695403

[38] Majak W Quinton DA Douwes HE Hall JW Muir AD. The effect of clipping on the growth and miserotoxin content of Columbia milkvetch. J Range Manage. (1988) 41:26–9. 10.2307/3898785

[39] Zhang ZW Wang YL Wang WK Li YH Cao ZJ Li L. The inhibitory action mode of nitrocompounds on *in vitro* rumen methanogenesis: a comparison of nitroethane, 2-nitroethanol and 2-nitro-1-propanol. J Agric Sci. (2019) 157:471–9. 10.1017/S002185961900086830886898

[40] Duin EC Wagner T Shima S Prakash P Cronin B Yáñez-Ruiz DR. Mode of action uncovered for the specific reduction of methane emissions from ruminants by the small molecule 3-nitrooxypropanol. Proc Natl Acad Sci U S A. (2016) 113:6172–7. 10.1073/pnas.1600298113 27140643 PMC4896709

[41] Majak W. Further enhancement of 3-nitropropanol detoxification by ruminal bacteria in cattle. Can J Anim Sci. (1992) 72:863–70. 10.4141/cjas92-0983710925

[42] Wolfensohn S Lloyd M. Chapter 12: The larger domestic species. In: Handbook of Laboratory Animal Management and Welfare, 3rd Edn. Oxford: Blackwell Publishing Ltd (2003). p. 326–64.

[43] Majak W Pass MA Muir AD Rode LM. Absorption of 3-nitropropanol (miserotoxin aglycone) from the compound stomach of cattle. Toxicol Lett. (1984) 23:9–15. 10.1016/0378-4274(84)90003-1 6435289

[44] Pass MA Majak W Muir AD Yost GS. Absorption of 3-nitropropanol and 3-nitropropionic acid from the digestive system of sheep. Toxicol Lett. (1984) 23:1–7. 10.1016/0378-4274(84)90002-X 6485010

[45] Hristov AN Oh J Giallongo F Frederick TW Harper MT Weeks HL. An inhibitor persistently deceased enteric methane emissions from dairy cows with no negative effect on milk production. Proc Natl Acad Sci U S A. (2015) 112:10663–8. 10.1073/pnas.1504124112 26229078 PMC4553761

[46] Melgar A Harper MT Oh J Giallongo F Young ME Ott TL. Effects of 3-nitrooxypropanol on rumen fermentation, lactational performance, and resumption of ovarian cyclicity in dairy cows. J Dairy Sci. (2020) 103:410–32. 10.3168/jds.2019-17085 31733848

[47] Myhre G Shindell D Bréon Bréon F-M Collins W Fuglestvedt J Huang J. Anthropogenic and natural radiative forcing. In: Stocker TF, Qin D, Plattner G-K, Tignor M, Allen SK, Doschung J, et al. editors. Climate Change 2013: The Physical Science Basis. Contribution of Working Group I to the Fifth Assessment Report of the Intergovernmental Panel on Climate Change Cambridge: Cambridge University Press (2013). p. 659–740.

[48] Griffin TB Stein AA Coulston F. Chronic inhalation exposure of rats to vapors of nitroethane. Ecotoxicol Environ Saf. (1988) 16:11–24. 10.1016/0147-6513(88)90012-7 3181065

[49] Alemzadeh I Sakhaei M. Enzymatic synthesis of theanine in the presence of L-glutaminase produced by *Tricoderma koningii*. Appl Food Biotechnol. (2017) 4:113–21. 10.22037/afb.v4i2.15713

[50] Chi M-C Lin M-G Huang Y-F Chen Y-Y Wang T-F Lin L-L. Enzymatic synthesis of L-theanine from L-glutamine and ethylamine by *Bacillus licheniformis* γ-glutamyltranspeptidase and its mutants specialized in transpeptidase activity. Biocatal Agric Biotechnol. (2019) 22:101393. 10.1016/j.bcab.2019.101393

[51] Looper CG Stallcup OT Reed FE. Deamination of amino acids *in vivo* by rumen microorganisms. J Anim Sci. (1959) 18:954–8. 10.2527/jas1959.183954x

[52] Anderson RC Majak W Rasmussen MA Allison MJ. Detoxification potential of a new species of ruminal bacteria that metabolizes nitrate and naturally occurring nitrotoxins. In: Garland T, Barr AC, editors. Toxic Plants and Other Natural Toxicants. New York, NY: CAB International (1998). p. 154–8.

